# Enhanced detection of aromatic oxidation products using NO_3_^−^ chemical ionization mass spectrometry with limited nitric acid†

**DOI:** 10.1039/d4ea00087k

**Published:** 2024-10-15

**Authors:** Olga Garmash, Avinash Kumar, Sakshi Jha, Shawon Barua, Noora Hyttinen, Siddharth Iyer, Matti Rissanen

**Affiliations:** a Aerosol Physics Laboratory, Physics Unit, Faculty of Engineering and Natural Sciences, Tampere University FI-33720 Tampere Finland matti.rissanen@tuni.fi; b Department of Chemistry, University of Copenhagen DK-2100 Copenhagen Denmark olga.garmash@chem.ku.dk; c Atmospheric Research Centre of Eastern Finland, Finnish Meteorological Institute FI-70211 Kuopio Finland; d Department of Chemistry, University of Helsinki FI-00014 Helsinki Finland

## Abstract

Nitrate ion-based chemical ionization mass spectrometry (NO_3_^−^-CIMS) is widely used for detection of highly oxygenated organic molecules (HOMs). HOMs are known to participate in molecular clustering and new particle formation and growth, and hence understanding the formation pathways and amounts of these compounds in the atmosphere is essential. However, the absence of analytical standards prevents robust quantification of HOM concentrations. In addition, nitrate-based ionization is usually very selective towards the most oxygenated molecules and blind to less oxygenated compounds hindering the investigation of molecular formation pathways. Here, we explore varying concentrations of nitric acid reagent gas in the sheath flow of a chemical ionization inlet as a method for detecting a wider range of oxidation products in laboratory-simulated oxidation of benzene and naphthalene. When the concentration of reagent nitric acid is reduced, we observe an increase in signals of many oxidation products for both precursors suggesting that they are not detected at the collision limit. The sensitivity of naphthalene oxidation products is enhanced to a larger extent than that of benzene products. This enhancement in sensitivity has a negative relationship with molecular oxygen content, the oxygen-to-carbon ratio, the oxidation state of carbon, and lowered volatility. In addition, the sensitivity enhancement is lower for species that contain more exchangeable H-atoms, particularly for accretion products. While more experimental investigations are needed for providing the relationship between enhancement ratios and instrumental sensitivities, we suggest this method as a tool for routine check of collision-limited sensitivities and enhanced detection of lower-oxygenated species.

## Abstract 2

With limited concentration of reagent nitric acid (HNO_3_) in the nitrate ion-based chemical ionization mass spectrometry (NO_3_^−^-CIMS) we observed increased sensitivity towards many of the aromatic oxidation products.

## Graphical abstract

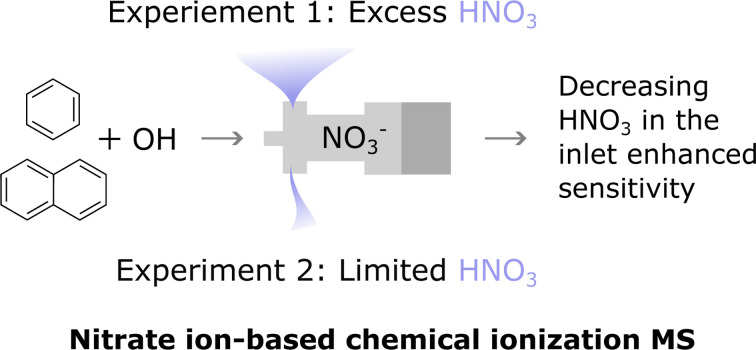

**Environmental significance**

Nitrate-ion chemical ionization mass spectrometry is extensively used in atmospheric science to detect highly oxygenated organic molecules (HOMs). Quantifying HOMs in different environments is of utmost importance in understanding their role in aerosol particle formation. However, calibration standards are lacking and often a single calibration factor is used for quantifying a large range of these compounds. Here, we experimentally show that this approach will underestimate HOM yield from naphthalene oxidation at least by a factor of 4. In addition, by modifying conditions in the ionization region, we show that the nitrate ionization method is also applicable to detecting less oxygenated compounds and peroxy radicals which enhances our ability to determine HOM formation pathways.

## 1 Introduction

Highly oxygenated organic molecules (HOMs) are recognized as central to closing gaps in our knowledge of secondary organic aerosol (SOA) formation in the atmosphere. They form in oxidation of volatile organic compounds (VOCs) under atmospheric conditions through a chain reaction called autoxidation mediated by peroxy radicals and defined as compounds with 6 or more oxygen atoms.^1^ HOMs form rapidly in the gas phase oxidation of biogenic VOCs, such as different monoterpenes, but also other alkenes,^2–5^ and more recently were discovered to form also from anthropogenic emissions, such as aromatics and alkanes.^6–10^ As autoxidation progresses, formed HOMs become more functionalized and less volatile. As a result, HOMs are able to condense efficiently even on the smallest particles in the atmosphere.^2,11,12^

The detection of HOMs largely relies on nitrate chemical ionization coupled to a time-of-flight mass spectrometer known as NO_3_^−^-CI-APi-TOF^13^ or NO_3_^−^-CIMS.^14^ This technique relies on the production of (HNO_3_)_0–2_NO_3_^−^ reagent cluster ions in the ionization region and subsequent formation of NO_3_^−^ adducts with the sample molecules. In normal operation with nitric acid excess, the adduct formation in the NO_3_^−^ ionization scheme is selective: based on quantum chemical calculations, an organic molecule should have at least two strong hydrogen bonding functionalities, such as hydroxy (–OH) or hydroperoxy (–OOH) groups, to be detected with high sensitivity.^15^

Several configurations of NO_3_^−^ chemical ionization inlets have been developed. One of the commonly used inlets is an Eisele-type inlet where a sheath flow carrying NO_3_^−^ ions is co-axially introduced around the sample flow.^14^ This results in a near wall-less system. Reagent ions are then directed towards the sample using an electrical field, where they are allowed to react. In commercially available Eisele-type NO_3_^−^ inlets (based on Jokinen *et al.*^13^), the reaction time is in the order of 110–160 milliseconds.^16,17^ Another possible set-up is a cross-flow ionization inlet (cluster CIMS) where ions are introduced perpendicularly to the sample flow.^18^ Recently, also a third configuration was introduced, a multi-scheme chemical ionization inlet (MION). In this set-up, reagent ions are injected perpendicularly to the sample flow, while neutral nitric acid is pulled away to the exhaust.^16,19^ In this study, we investigate the performance of the Eisele-type co-axial inlet as the most commonly used nitrate-based inlet currently in our field and we use NO_3_^−^-CIMS notation throughout the text.

The benefit of utilizing CIMS is ultimately dependent on our ability to quantify molecules of interest. However, no analytical HOM calibration standard currently exists. When HOMs were discovered, Ehn *et al.*^2^ calculated that HOM ion–molecule collision rates with nitrate ion adducts were similar to that of sulfuric acid. Therefore, sulfuric acid calibration^20^ has been routinely used to determine the HOM sensitivities. As a result, concentrations and yields of HOMs are regularly reported as lower limit estimates,^2^ especially since it became clear that they may be detected at lower sensitivities if they lack strong hydrogen bonding groups.^15^ Sometimes, correction factors are applied to account for reduced sensitivity of species with higher volatility.^11^ Quantification of HOMs remains one of the largest challenges in current studies that aim to elucidate the formation pathways of SOA.

The detection of organic sample molecules in NO_3_^−^ ionization through adduct formation proceeds in competition with neutral nitric acid (HNO_3_). NO_3_^−^ forms very stable clusters with HNO_3_, and that is why NO_3_^−^ ionization is selective towards highly functionalized species that can bind more strongly to NO_3_^−^ than to HNO_3_.^15^ As a result, the sensitivity of a given species is a function of the concentration of neutral HNO_3_ in the ionization region.^15^ Neutral HNO_3_ in the ionization region can originate from the sample flow or alternatively is carried by the reagent ions and released during the ligand exchange reaction with sample molecules.^21^ Based on recent flow simulations in the CI-inlet, a very minor fraction of neutral HNO_3_ will diffuse from sheath to sample flow.^17^ As demonstrated by quantum chemical computations, the sensitivity remains constant as a function of nitric acid concentration (provided that the total number of ions remains constant) if a molecule is detected at the collision limit, *i.e.* maximum sensitivity.^15^

Not all organic oxidation products that NO_3_^−^-CIMS detects can be classified as HOMs. For instance, NO_3_^−^-CIMS can detect four and five oxygen-containing products in aromatic oxidation systems,^8^ while in α-pinene ozonolysis, detected species primarily have 6 or more oxygen atoms.^2,22^ NO_3_^−^-CIMS can also detect 4-nitrophenol, a compound containing only three O-atoms, as the binding of the 4-nitrophenol–nitrate adduct is comparable to that of the nitric acid–nitrate adduct.^21^ Even at low sensitivities, however, the detection of less oxygenated species in laboratory studies, which are generally present at considerably higher concentrations than HOMs, would greatly enhance the applicability of NO_3_^−^-CIMS.

In our current study, we probe the ability of NO_3_^−^-CIMS to detect oxidation products of benzene and naphthalene, including HOMs and less oxygenated products. We do so by running laboratory experiments with excess and deficit of neutral HNO_3_ in the sheath flow within the chemical ionization inlet. We revisit the concepts introduced by Hyttinen *et al.*^15^ from an experimental perspective and propose varying HNO_3_ concentrations in the sheath flow as a method for detecting less oxygenated RO_2_ radicals and closed-shell species as well as for verifying the applicability of collision-limited sensitivities. Our study demonstrates the extended usability of the nitrate ionization scheme to produce a more complete understanding of HOM formation pathways in the laboratory and atmosphere.

## 2 Methods

We conducted oxidation experiments in a borosilicate flow reactor with a residence time of 14.5 seconds. We used benzene and naphthalene as precursor VOCs. Benzene (BEN, C_6_H_6_) was fed from a prepared gas bottle while naphthalene (NPT, C_10_H_8_) was introduced to the reactor by flowing cryogenic nitrogen (N_2_) over a pure solid naphthalene sample. The oxidant hydroxyl radical (OH) was produced in the dark oxidation of tetramethylethylene (TME) by ozone. Table 1 lists the conditions for the conducted experiments: three experiments with naphthalene and one with benzene. With naphthalene, experiment (exp.) 1 was performed at the lowest OH production rate and is, therefore, referred to as the low-RO_2_ radical experiment. Experiments 2 and 3, on the other hand, test the reproducibility of the method at higher RO_2_ radical production rates. Note that the high RO_2_ radical experiment refers to the higher production of oxidation products and usually results in a lower detected RO_2_ concentration as bimolecular termination reactions dominate. With benzene (exp. 4), the concentration of the OH radical was adjusted only for the higher RO_2_ production regime. The concentrations were controlled at about 72 and 108 parts per billion volume (ppbv) of TME and 41–382 ppbv of ozone, resulting in 1.5, 9.8 and 14.3 ppbv of reacted VOCs in exp. 1, 2–3 and 4 respectively.

**Table 1:** The experimental conditions for HNO_3_ excess and deficit runs for naphthalene and benzene experiments. Experimental conditions for exp. 2 and 3 are near identical. The reacted VOC was calculated using bimolecular reaction rate coefficients *k*_OH–NPT_ of 2.17 × 10^−11^, *k*_OH–BEN_ of 1.28 × 10^−12^, *k*_O_3_–TME_ of 1.5 × 10^−15^, *k*_OH–TME_ of 1 × 10^−10^ and *k*_OH–O_3__ of 7.3 × 10^−14^ cm^3^ s^−1^,^23^ accounting for the corresponding reactions and flow reactor residence time of 14.5 s; secondary oxidation reactions were not considered. *Q* is flow and *r* is the sensitivity enhancement ratio

#	*Q*_HNO_3__, sccm	VOC	RO_2_ regime	Total ion count, cps	O_3_, ppbv	TME, ppbv	VOC, ppmv	VOC reacted, ppbv	Total product signal, cps	RO_2_ fraction, %	Dimer fraction, %	*r*_HOM_ O_≥6_
1	0	C_10_H_8_	Low	20 930	41	72	6.9	1.5	150	11	42	3.9
	10	C_10_H_8_	Low	21 278	41	72	6.9	1.5	40	8.8	60	
2	0	C_10_H_8_	High	19 895	288	72	6.9	9.8	2100	2.4	39	4.0
	10	C_10_H_8_	High	20 920	288	72	6.9	9.8	520	2.5	57	
3	0	C_10_H_8_	High	21 088	288	72	6.9	9.8	2500	1.9	39	2.3
	10	C_10_H_8_	High	21 989	288	72	6.9	9.8	1040	2.2	54	
4	0	C_6_H_6_	High	22 024	382	108	22	14.3	1800	1.3	7.4	1.3
	10	C_6_H_6_	High	23 735	382	108	22	14.3	1000	1.5	9.9	

**Fig. 1:**
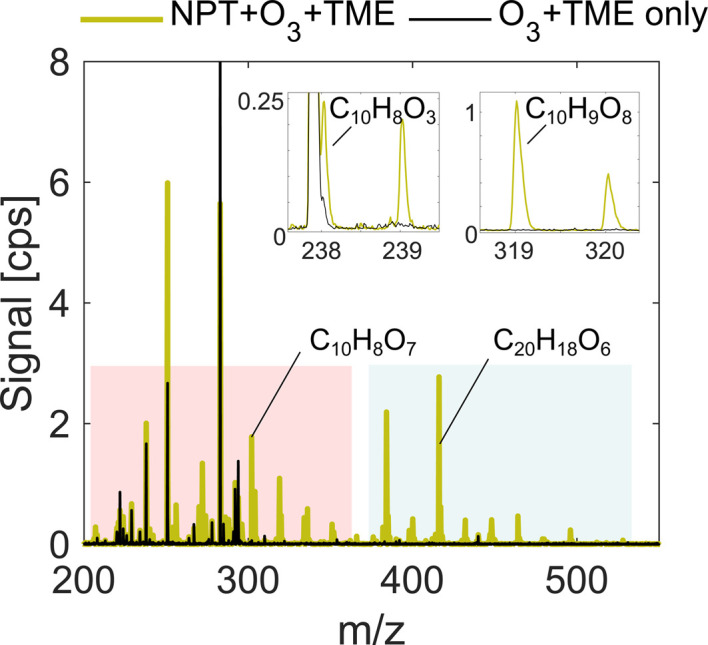
Comparison of spectra for naphthalene oxidation (green) and background experiments (black). The presented experiment is low RO_2_ run (exp. 1) with deficit of nitric acid. The pink shaded area indicates the monomer region while the blue area shows the accretion product region. NPT stands for naphthalene, while TME stands for tetramethylethylene. The signal is reported directly in measured counts per second (cps). The high background peaks in black are iodine-containing contaminants in the flow reactor, which are used for mass axis calibration.

The oxidation products were sampled using an Eisele-type NO_3_^−^ inlet (Fig. S1†).^13,14^ The inlet was coupled to an atmospheric pressure interface time-of-flight mass spectrometer, APi-TOF.^24^ In excess HNO_3_ experiments (normal operation), nitric acid was directed into the sheath flow by flowing 10 standard cubic centimeters per minute (sccm) cryogenic N_2_ over the liquid surface, which mixed into 20 standard liters per minute (slpm) sheath flow. The experiments with a low nitric acid concentration (deficit) were conducted by setting the N_2_ flow over nitric acid to 0 sccm. In that case, nitric acid entered sheath flow through diffusion from the vial and the inlet walls. The deficit of nitric acid was recorded when we observed the change in the reagent ion concentration and distribution as well as the appearance of the carbonic acid–nitrate ion adduct (CH_2_O_3_NO_3_^−^) at a nominal 124 mass-to-charge ratio (*m*/*z*) (see section 3.1). Flow reactor bath gas and sheath flow was supplied *via* a clean air generator (AADCO-737-15). Ozone was produced by photolyzing the clean air using a mercury lamp (UVP, Analytik Jena). The concentration of ozone was measured using an ozone analyzer (2B Technologies model 205), while gas-bottle mixing ratios of TME and benzene were determined by pressure gauge measurement in the gas mixing set-up. NO_3_^−^-CIMS data were processed using the tofTools v6.12 package for MATLAB. Peaks were identified by high resolution peak fitting expanding upon previous studies^7,8^ (see Tables S1–S3†). Ions of interest were determined by comparing oxidation and background experiments (as shown in Fig. 1) in the presence and absence of VOCs, respectively. The analysis solely focuses on compounds that were detected as adducts with NO_3_^−^, which is omitted from all oxidation product formulae within the text and figures. In addition, only compounds that lost no more than one carbon atom are studied (C_≥5_ for benzene and C_≥9_ for naphthalene). Smaller fragmentation products, which are minor fractions of detected products, are not considered.

Our analysis takes place in absolute units of the detected signal, counts per second (cps), and normalization to the reagent ion concentration is omitted as deficit of nitric acid introduces non-linear effects on ionization efficiency of sample molecules. However, the total ion count (TIC) remains near constant at 2.0–2.4 × 10^4^ cps as shown in Table 1. It is also important to note that some individual ions of interest had as low concentrations as 0.1–1 cps, below 0.005% of TIC (Fig. 1) depending on experimental conditions. The sensitivity enhancement is calculated as a ratio between the signal for an individual ion in HNO_3_ deficit and HNO_3_ excess experiments and is referred to as the sensitivity enhancement ratio (*r*).

In order to verify the amount of available H-bond donor groups, we also conducted experiments where heavy water (D_2_O) was added. The D_2_O (Eurisotop, 99.96%) was only added to HNO_3_ deficit experiments where few droplets of heavy water were evaporated from a glass bubbler into the flow reactor. As the system was dry otherwise, a near-complete conversion of all exchangeable H-atoms was expected and confirmed *via* monitoring HNO_3_NO_3_^−^ fully converting to DNO_3_NO_3_^−^ (Fig. S4†). Table 2 presents the conditions for D_2_O-addition experiments, with some small differences to the experiments presented in Table 1. The addition of D_2_O also introduced new contaminants to the system, so the signal is corrected for background (VOC is turned off).

**Table 2:** D_2_O addition experiments. D_2_O addition is indicated with 1 (added) or 0 (not added)

#	*Q*_HNO_3__, sccm	VOC	D_2_O addition	Total ion count, cps	O_3_, ppbv	TME, ppbv	VOC, ppmv	VOC reacted, ppbv
5	0	C_10_H_8_	0	23 070	40	18	1.7	0.36
	0	C_10_H_8_	1	22 328	40	18	1.7	0.36
6	0	C_6_H_6_	0	21 879	382	18	5.4	2.7
	0	C_6_H_6_	1	21 389	382	18	5.4	2.7

The saturation concentration (proportional to volatility) of each oxidation product at 300 K is calculated using 2D volatility basis set 2D-VBS^25^ as follows:
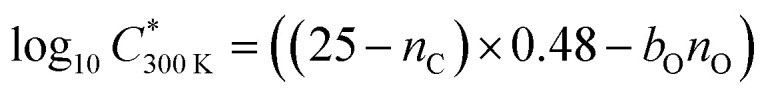
where *n*_C_ is the number of carbon atoms, *n*_O_ is the number of oxygen atoms, and *b*_O_ is the coefficient describing the average effect of each added oxygen atom on log_10_ C*. A *b*_O_ of 1.72 and 1.55 was used for naphthalene and benzene respectively following empirical determination by Wang *et al.*^26^ This approach was confirmed to work the best for estimating volatilities of aromatic oxidation products.^26^ The oxidation state of carbon in Fig. 6b was calculated following a simple approximation by Kroll *et al.*,^27^ as OSc = 2*n*_O_:*n*_C_ − *n*_H_/*n*_C_, where *n*_O_, *n*_C_ and *n*_H_ are numbers of oxygen, carbon and hydrogen atoms respectively.

The following method was used to calculate the binding enthalpies of the oxidation products of benzene and naphthalene with NO_3_^−^. Conformer search to generate the possible input geometries was carried out using the Merck Molecular Force Field (MMFF) method using the Spartan '20 program.^28^ All initial geometries were first optimized at the B3LYP/6-31+G* level of theory, followed by optimizations and frequency calculations at the ωB97X-D/aug-cc-pVTZ level of theory on conformers with relative electronic energies within 2 kcal mol^−1^ of the global minimum. These geometry optimizations were performed using the Gaussian 16 program.^29^ The domain-based local pair natural orbital coupled cluster method (DLPNO-CCSD(T)) with the aug-cc-pVTZ basis set was used to compute the single-point electronic energy corrections on the global minima geometries. These were carried out using the ORCA program (version 4.2.1).^30^

## 3 Results and discussion

### 3.1. Monitoring the change in the HNO_3_ concentration

**Fig. 2:**
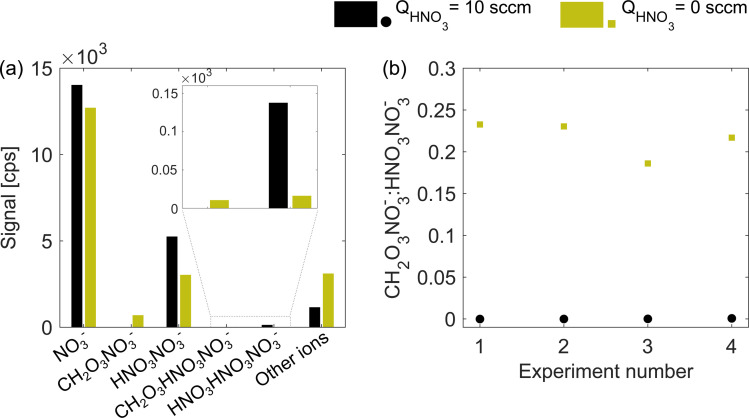
Distribution of ions in excess (*Q*_HNO_3__ = 10 sccm, black) and deficit (*Q*_HNO_3__ = 0 sccm, green) of nitric acid. Panel (a) shows the ion signals in counts per second (cps) for naphthalene experiment 2, and panel (b) shows the ratio between the carbonic acid–nitrate adduct (CH_2_O_3_NO_3_^−^, *m*/*z* 124) and nitric acid dimer (HNO_3_NO_3_^−^, *m*/*z* 125) for all four experiments (see Table 1). The exact distribution and ratio will be specific to the instrument, settings and absolute concentration of neutral nitric and carbonic acids.

An adduct of carbonic acid CH_2_O_3_ and NO_3_^−^ located at 124 nominal mass-to-charge (*m*/*z*) can be used as an indicator for insufficient nitric acid in the chemical ionization inlet. At normal operation (excess of HNO_3_), carbonic acid is not detected and a sudden appearance of *m*/*z* 124 usually points at a fault in the HNO_3_ feeding line. In routine operation, a peak at *m*/*z* 124 is avoided while here we use it as a proxy for the HNO_3_ concentration under deficit conditions. Fig. 2a illustrates the absolute abundance of nitrate reagent ions (monomer NO_3_^−^, dimer HNO_3_NO_3_^−^, and trimer (HNO_3_)_2_NO_3_^−^) as well as CH_2_O_3_NO_3_^−^ and CH_2_O_3_HNO_3_NO_3_^−^ adducts for the naphthalene experiment 2 (see Table 1). When the HNO_3_ flow (*Q*_HNO_3__) was shut off, the total ion count remained nearly constant (1.99 × 10^4^ compared to 2.09 × 10^4^ cps) while the distribution of reagent ions changed. Absolute concentrations of the nitric acid monomer, dimer (*m*/*z* 125) and trimer decreased, while the concentration of carbonic acid–nitrate adducts increased. The increase in the signal of other ions demonstrates that more of the sample molecules became ionized. Fig. 2b shows that all four experiments had a very similar *m*/*z* 124-to-*m*/*z* 125 ratio during HNO_3_ deficit runs (green squares), with values between 0.18 and 0.24. Provided the stable carbonic acid concentration in the sheath flow, the *m*/*z* 124-to-*m*/*z* 125 ratio should be inversely proportional to the total concentration of HNO_3_ in the ionization region.

### 3.2. Enhanced detection of lower oxygenated species and RO_2_ radicals

**Fig. 3:**
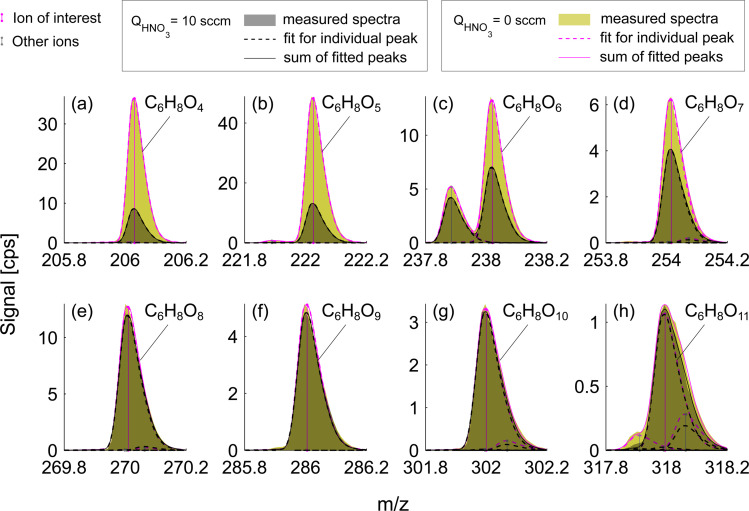
Mass spectra of oxidation products formed during benzene oxidation by OH (exp. 4). 10-minute averaged mass spectra in excess and deficit of HNO_3_ are depicted in black and green respectively. The difference between green and gray shaded areas represents the enhancement of the sensitivity due to the lowered HNO_3_ concentration. Dashed magenta curves show individual peak fits for the HNO_3_ deficit experiment (*Q*_HNO_3__ = 0 sccm) and black dashed curves – are for the HNO_3_ excess experiment (*Q*_HNO_3__ = 10 sccm). Vertical magenta lines indicate the position of the ion which is identified with a chemical formula (NO_3_^−^ is omitted) while other peaks are shown with gray vertical lines. The sum of all fitted peaks to the corresponding nominal mass is shown in solid magenta and solid black lines. All species shown in panels (a–h) are closed-shell molecules.

Experiments conducted at HNO_3_ deficit allowed for enhanced detection of less oxygenated closed-shell products and RO_2_ radicals. This effect is observed due to the reduced competition of sample molecules with nitric acid itself for the formation of an adduct with the nitrate ion, NO_3_^−^. Fig. 3 presents a mass spectrum of selected oxidation products in the benzene experiment. The enhancement in sensitivity is visible as the difference between signals for deficit and excess of HNO_3_ shown as green and gray shaded areas respectively. Compounds with the fewest oxygen atoms, C_6_H_6_O_4_ and C_6_H_6_O_5_, observe the highest enhancement in the signal, factors of 4.3 and 3.7 respectively (Fig. 3a and b). The enhancement is lower for C_6_H_8_O_6_ (1.9) and C_6_H_8_O_7_ (1.5) (Fig. 3c and d) switching to equal signals detected in both runs for higher oxygenated products C_6_H_8_O_8–11_ (Fig. 3e–h). This suggested that among these products, only C_6_H_8_O_8–11_ are detected at the collision limit, as their detected signals remain constant. However, it is clear that we are able to enhance sensitivity and the signal-to-noise ratio of other compounds.

**Fig. 4:**
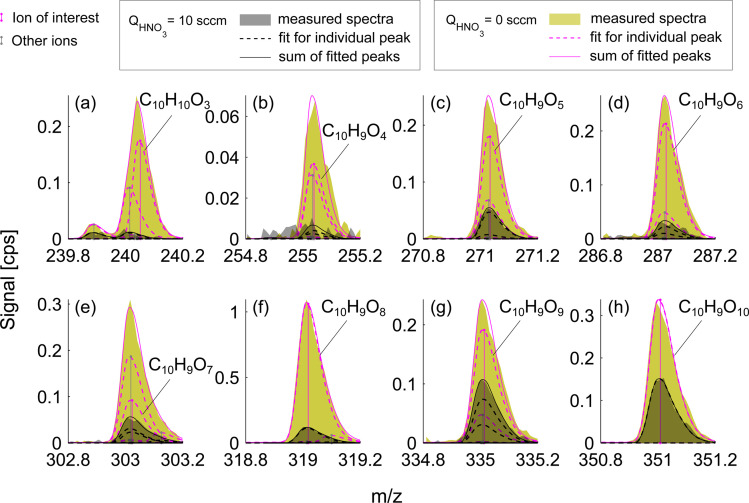
Mass spectra of oxidation products formed during naphthalene oxidation by OH in the low RO_2_ regime (exp. 1). The colors are the same as in Fig. 3. Panel (a) shows a closed-shell molecule while panels (b–h) show RO_2_ radicals that have 9 H-atoms.

This effect of enhanced sensitivity is even more pronounced in the naphthalene experiment with low VOC turnover and consequently lower oxidation product signals. Fig. 4 shows closed-shell product C_10_H_10_O_3_ and seven RO_2_ radicals with 4–10 oxygen atoms for exp. 1 (see Table 1). C_10_H_10_O_3_ and C_10_H_9_O_4_ become detectable under HNO_3_ deficit conditions, while C_10_H_9_O_6_ and C_10_H_9_O_9_ radicals become easier to separate from overlapping peaks. As shown in Fig. 4, most of the overlapping peaks are constrained isotope signals from −1 *m*/*z*. For instance, in Fig. 4b, a peak indicated in grey is a ^13^C isotope of C_10_H_8_O_4_. The sensitivity enhancement of the most abundant radical C_10_H_9_O_8_ was almost 10-fold, while for C_10_H_9_O_9_ and C_10_H_9_O_10_ an enhancement factor of 2.7 and 2.4 was observed, respectively, which is further discussed in sections 3.3 and 3.5. Fig. 3 and 4 demonstrate the enhanced ability of NO_3_^−^-CIMS to detect less oxygenated closed-shell products and RO_2_ radicals.

The main reason for the increased sensitivity in the HNO_3_ deficit experiment is the relative increase of the NO_3_^−^ monomer.^15^ In the ion source, lower neutral HNO_3_ causes a shift in the reagent ion cluster distribution towards more abundant free NO_3_^−^. With the increase in free NO_3_^−^, more sample molecules (M) are ionized *via* simple anion (NO_3_^−^) attachment, as opposed to ligand switching (with HNO_3_·NO_3_^−^) in normal operation. The probability of ligand switching depends exponentially on the binding energies of the M·NO_3_^−^ and HNO_3_·NO_3_^−^ clusters. Therefore, the enhancement in sensitivity is expected for all species, the binding energy of which with NO_3_^−^ is lower than the binding energy for the HNO_3_·NO_3_^−^ cluster, as shown by Hyttinen *et al.*^15^ Our experiments were especially suitable to observe this sensitivity increase as we used HNO_3_-free sample flow. High HNO_3_ concentrations in the sample flow relative to the reagent ion and sample concentration would inhibit this effect due to the sufficient number of HNO_3_ molecules for substituting the sample molecules in M·NO_3_^−^ clusters forming HNO_3_·NO_3_^−^.^15^ Therefore, the applicability of this method is limited to low HNO_3_ environments.

Our ability to enhance the sensitivity of NO_3_^−^-CIMS is especially important for studying the first steps of VOC autoxidation. In order to avoid excessive bimolecular reactions that can occur specifically in laboratory simulations,^31^ it is imperative to maintain low RO_2_ radical concentrations and shorten the reaction times. This approach often pushes the concentration of compounds of interest to levels below the detection limit of CIMS. This is confirmed when we compare exp. 2 and 3 with higher VOC turnover (and hence a higher initial RO_2_ concentration) to exp. 1 with lower VOC turnover. Exp. 1 has a lower total concentration of oxidation products (150 cps *vs.* 2100–2500 cps) and a higher fraction of RO_2_ radicals due to longer radical lifetime (11.1% compared to 2.4% and 1.9% in exp. 2 and 3, respectively). Moreover, some of the species shown in Fig. 4 were only detected in deficit of HNO_3_. As a result, running the instrument with lower HNO_3_ could be used as an approach for enhancing the sensitivity of NO_3_^−^-CIMS to less oxygenated species and even helping to discover new intermediates, similarly as recently achieved by Berndt.^32^

### 3.3. Relative sensitivity enhancement in benzene and naphthalene systems

**Fig. 5:**
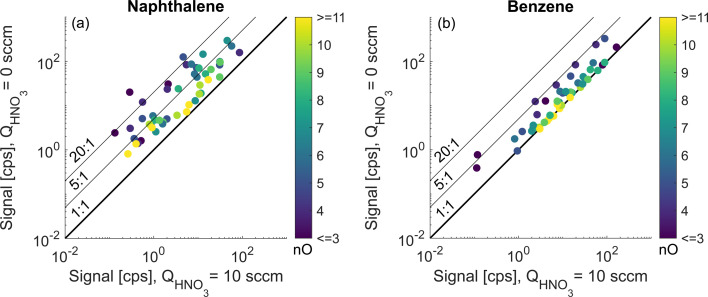
Comparison of sensitivity enhancement for oxidation products as a response to a decrease in the HNO_3_ concentration in the CI-inlet in the high RO_2_ regime between naphthalene (a) and benzene experiments (b) (exp. 2 and 4). The enhancement ratio is the slope on this figure, and color is the number of oxygen atoms (*n*_O_).

When projected to the whole array of observed oxidation products, the sensitivity enhancement introduced in the previous section is greater in the naphthalene oxidation system than in benzene. Fig. 5 compares the signals for each studied oxidation product at the deficit and excess of HNO_3_ in the sheath flow of the CI-inlet. Naphthalene high RO_2_ (exp. 2) and benzene high RO_2_ (exp. 4) experiments are chosen as they have the most similar experimental conditions (Fig. 2b). In Fig. 5, both monomers and dimeric accretion products are examined. While there is a correlation of the enhancement ratio with the number of oxygen atoms for the naphthalene oxidation products (Fig. 5a), none of the detected molecules appeared at the 1 : 1 line, which would correspond to no enhancement. In contrast, in the benzene system, roughly half of the products had no enhancement in sensitivity and the dependency on oxygen atom content is more pronounced (Fig. 5b and S3†). As a result, we conclude that a larger fraction of benzene than naphthalene oxidation products is detected at the collision limit.

While it appears that almost no products in naphthalene systems are detected at the collision limit, many of the species have an enhancement ratio below 2 (Fig. S2†). The lowest enhancement ratios are observed for the following products: C_9_H_8_O_7_ (1.5), C_10_H_8_O_8_ (1.6), C_10_H_8_O_9_ (1.4), C_10_H_9_O_10_ (1.8), C_10_H_10_O_10_ (1.7), C_20_H_18_O_4_ (1.8), C_20_H_18_O_12_ (1.7), and C_20_H_18_O_13_ (1.3). Beside C_20_H_18_O_4_, all these products can be classified as HOMs based on their oxygen content.^1^ While the general trend in the increased sensitivity is reproducible between the three naphthalene experiments, one of the high RO_2_ experiments (exp. 3) exhibits smaller enhancements for individual ions (Fig. S2†). This could be explained by a lower *m*/*z* 124-to-*m*/*z* 125 ratio (Fig. 2b) indicating that the HNO_3_ concentration in the sheath flow under deficit conditions of this experiment was higher than that in the other experiments. Fig. S2† also shows distinct enhancement ratio profiles as a function of the specific elemental composition. This information could provide an insight into the type and number of functional groups or a specific formation pathway for a given oxidation product, though it is outside the scope of this work and only touched briefly upon in section 3.4.

In contrast to naphthalene, most of the products in benzene oxidation with 7 or more O-atoms were detected at the apparent collision limit (Fig. 5b). The sensitivity of all ions but one (C_6_H_6_O_1_) increased by a factor of 5 or less, in comparison to many ions with an enhancement ratio of above 5 for naphthalene (Fig. 5a). Fig. S3† shows a detailed pattern for all benzene oxidation products detected. Interestingly, some of the less oxygenated species (*e.g.*, C_5_H_6_O_2_ (1.3), C_6_H_6_O_3_ (1.0) and C_6_H_8_O_3_ (1.5)) had a lower sensitivity enhancement than their intermediately oxygenated counterparts (*e.g.* C_6_H_6,8_O_4_, *r* ≈ 4). This is similar to some of the naphthalene oxidation products (Fig. S2†) and could be possibly explained by multiple oxidation steps that add 2 or more hydroxy (–OH) groups allowing the formation of stable adducts with the nitrate ion while the oxygen content remains low. The strong dependence of the binding strength on the exact geometry of the target compound was also investigated computationally and is presented below. It was not possible to elucidate the number of exchangeable H-atoms (section 3.4) for less oxygenated compounds in the benzene system due to interference from overlapping ions, and, therefore, they are not discussed further.

**Fig. 6:**
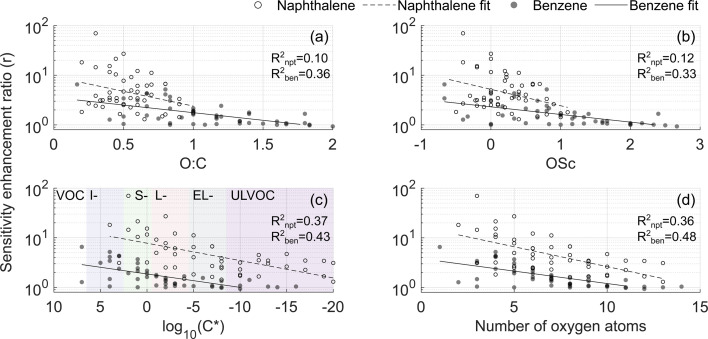
Sensitivity enhancement ratio as a function of (a) O : C, (b) OSc, (c) base 10 logarithm of saturation concentration in μg m^−3^, and (d) number of oxygen atoms. In this figure, experiments 2 and 4 are compared (Table 1). Regression coefficients are listed in Table S4.† In panel (c), the following notations are used: VOC for volatile organic compounds, I- for intermediate volatility, S- for semi low volatility, L- for low volatility, EL- for extremely low volatility, and ULVOC for ultra-low volatility organic compounds (VOC, IVOC, SVOC, LVOC, ELVOC, and ULVOC, respectively).

The dependence of the enhancement ratio (*r*) on different molecular characteristics is presented in Fig. 6. A statistically significant negative relationship of *r* is observed with the oxygen-to-carbon ratio (O : C), the oxidation state of carbon (OSc), lowered volatility (positive relationship with the saturation concentration C*) and the number of oxygen atoms. O : C and OSc account for differences in the amount of both carbon and oxygen atoms though they explain *r* in the naphthalene experiment the least (*R*^2^ = 0.1 and 0.12, Fig. 6a and b). In the naphthalene system, *r* is best explained by log_10_(C*) and the number of oxygen atoms (*R*^2^ = 0.37 and 0.36, Fig. 6c and d), in line with the finding by Hyttinen *et al.* (2018)^21^ that binding enthalpies correlate well with the number of O-atoms. For the benzene experiment, all parameters perform similarly, with the number of O-atoms being the best predictor of *r* (*R*^2^ = 0.48, Fig. 6d). The slopes of the linear models in Fig. 6 are similar between the two VOC systems, and higher *r* in the naphthalene experiment is further evident. Strikingly, even compounds classified as ultra-low volatility organic compounds (ULVOC) in naphthalene experiments are not detected at the collision limit, which suggests that the detection of naphthalene oxidation products is hindered in NO_3_^−^-CIMS to a larger extent than previously understood. Some of the reasons could be that benzene has a larger fraction of open-ring products eliminating steric constraints in adduct formation or more functional groups available for creating strong H-bonds.

Another plausible reason explaining why benzene oxidation products bind more strongly to NO_3_^−^ than naphthalene products is the differences in the molecular size. It is reasonable to assume that large compounds, such as C_10_H_10_O_8_ or C_20_H_16_O_10_, among others, would have at least two hydroxy or hydroperoxy functional groups needed for forming H-bonds with the nitrate ion. However, if these groups are located on the opposite sides of the molecule, the probability of forming H-bonds with a small NO_3_^−^ ion is reduced. This is confirmed by our quantum chemical calculations for a series of model dihydroxy-benzenes and naphthalenes (Table 3). Catechol, having *ortho* substituted hydroxy groups on a benzene ring, has the highest binding enthalpy with the nitrate ion, 26.2 kcal mol^−1^. The binding enthalpy decreases as the OH-groups are further apart: 23.1 kcal mol^−1^ for *meta* and 21.7 kcal mol^−1^ for *para* substitutions. The same pattern is observed for dihydroxynaphthalene: 1,2-dihydroxynaphthalene (*ortho*) has a binding enthalpy with nitrate of 30.6 kcal mol^−1^ which decreases to 23.8 kcal mol^−1^ for 1,4-dihydroxynaphthalene (*para*). It is worth noting that the binding enthalpy with nitrate is further reduced if the OH groups are substituted onto different aromatic rings, though the decrease is smaller than the *ortho* to *para* decrease. While the computational results confirm that the exact positions of the H-bond-donor groups on the molecules matter for the binding enthalpy and, hence, sensitivity, dihydroxynaphthalenes bind more strongly to nitrate than dihydroxybenzenes. This is in contrast to what we see in our experiments (Fig. 5) and suggests that the real oxidation products are very different from these model compounds causing better detection of benzene oxidation products compared to naphthalene. In addition to the size of the oxidation products and the relative position of the functional groups, the number of functional groups available for H-bonding may also play a role and is investigated in the following section.

**Table 3:** Binding enthalpies of model benzene and naphthalene-derived dihydroxy compounds with the nitrate ion

System		Binding enthalpy (Δ*H* kcal mol^−1^) with NO_3_^−^
**Benzene-derived**		
1,2-Dihydroxybenzene	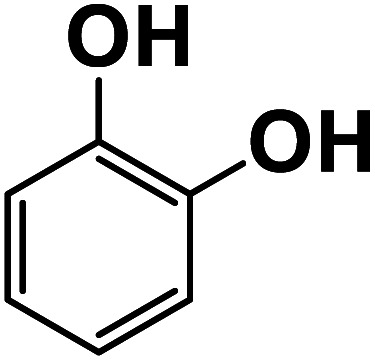	26.2
1,3-Dihydroxybenzene	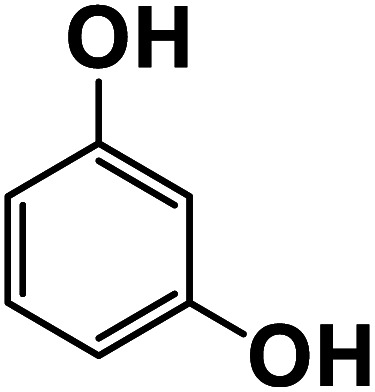	23.1
1,4-Dihydroxybenzene	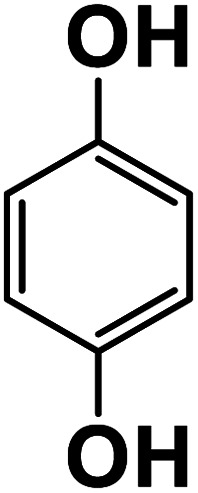	21.7

**Naphthalene-derived**		
1,2-Dihydroxynaphthalene	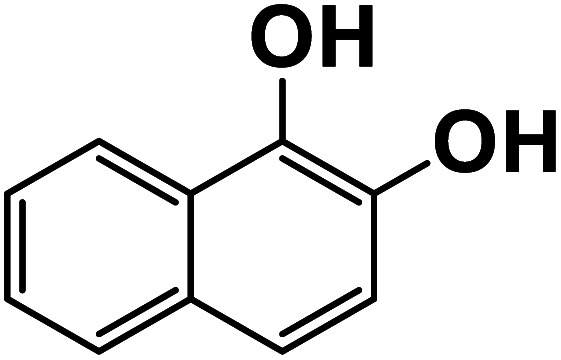	30.6
1,4-Dihydroxynaphthalene	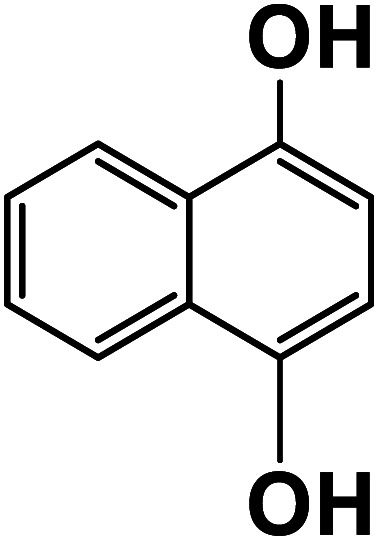	23.9
2,6-Dihydroxynaphthalene	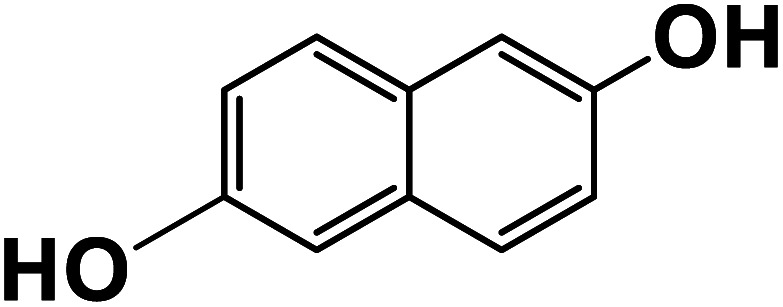	23.5

### 3.4. Relating enhancement ratio to the number of exchangeable H

**Fig. 7:**
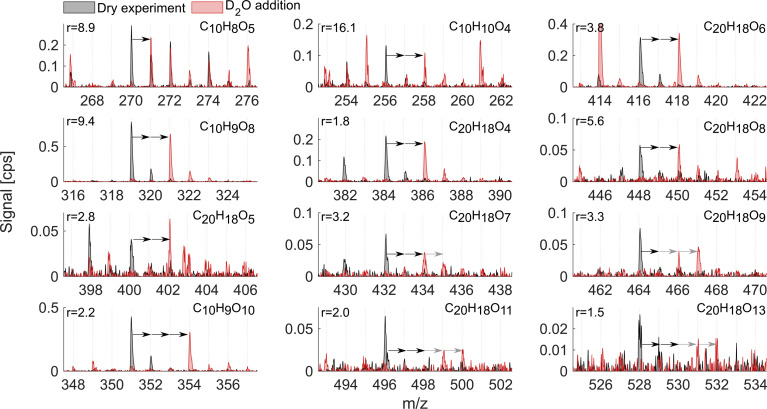
D_2_O addition experiment in the naphthalene oxidation system. The spectrum for the dry experiment is shown in black while the spectrum for D_2_O addition is shown in red. The arrows indicate the shift in the peak position as a response to H-atoms being substituted by heavy hydrogen, D-atoms. The origin of the first arrow shows the product of interest, the composition of which is marked on each subplot, while the number of arrows indicates the observed number of exchangeable hydrogens. Grey arrows indicate that the ion signal got split into several ions with different D-atoms. Near complete H → D conversion was achieved in this experiment, which was verified by the shift of the HNO_3_NO_3_^−^ reagent ion into DNO_3_NO_3_^−^. Reported *r* values are from exp. 1.

**Fig. 8:**
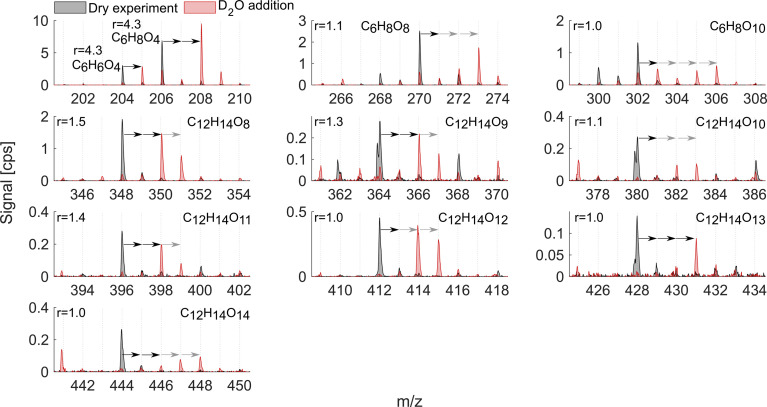
D_2_O addition experiment in the benzene oxidation system. The colors and notations are as in Fig. 7. The ions C_12_H_14_O_9_, C_12_H_14_O_10_, and C_12_H_14_O_13_ have overlapping peaks with negative mass defects at the corresponding *m*/*z* 364, 380, and 428. Reported *r* is from exp. 4.

As mentioned above, it is known that at least two strong H-bond donor groups are needed to detect a compound with high sensitivity as an adduct with NO_3_^−^ in NO_3_^−^-CIMS. One way to estimate the number of such H-bond donor groups is to determine the number of exchangeable hydrogens in the molecule. Fig. 7 and 8 show D_2_O addition experiments in which exchangeable H-atoms get replaced with deuterium, D, for the naphthalene and benzene systems, respectively. As a result, by monitoring the shift in the ion's mass, we can estimate the number of functional groups that can form hydrogen bonds with the nitrate ion.

When analyzing D_2_O experiments, some potential biases should be kept in mind. For instance, neighboring peaks that can also exchange H-atoms to D-atoms may overlap with the compound of interest. As a result, the products that are shown in Fig. 7 and 8 are selected based on the minimal interference from other compounds found at smaller *m*/*z*. If the ion's signal during D_2_O addition is split between two or more D-containing ions at different *m*/*z*, it is most plausible to conclude that several structural isomers are formed in the dry system. The following analysis compares the sensitivity enhancement ratios in both VOC systems with the number of exchangeable hydrogens.

The naphthalene experiment (Fig. 7) shows a somewhat inconsistent trend in the monomer region between the enhancement ratio (marked here as *r*) and the number of exchangeable hydrogens. Most of the products that reliably stood out exchanged 2 H-atoms with *r* ranging from 16.1 to 1.8 and no relationship to the number of O-atoms. C_10_H_8_O_5_ mostly exchanged only one H and its sensitivity enhanced as a result of HNO_3_ reduction by a factor of 9. This shows that NO_3_^−^-CIMS can also be suitable to detect species with only one OH/OOH group, though with much reduced sensitivity, and thus the modified HNO_3_ deficit set-up is especially useful. This is in contrast to the widely accepted two groups minimum requirement under normal CI-inlet operation (as understood from Hyttinen *et al.*^15^). Many of the HOM dimeric accretion products, C_20_H_18_O_7_, C_20_H_18_O_9_, C_20_H_18_O_10_, C_20_H_18_O_11_, and C_20_H_18_O_13_, exchanged more than 2 hydrogens, on average from 2.5 to 3.5, which was consistent with the increase in the number of O-atoms (7 to 13) and decrease in the enhancement ratio (3.3 to 1.5). Notably, even though these dimers would have 3 or 4 groups with available H-atoms to bind with NO_3_^−^, they are not detected at the collision limit suggesting that the large size of these C_20_ molecules with H-bond donating groups being further apart may hinder the clustering probability. Alternatively, C_20_ could lose the clustering ability with NO_3_^−^ due to strong internal hydrogen bonding, which would decrease their detection sensitivity.

The oxidation products in the benzene system (Fig. 8), as in naphthalene, exchanged one to four hydrogen atoms. However, the sensitivity enhancement was much lower. For instance, C_6_H_6_O_4_ exchanged 1 H and its *r* was 4.3, while C_10_H_8_O_5_ also exchanged 1 H in the naphthalene experiment (Fig. 7) but its *r* was 8.9. Monomer C_6_H_8_O_8_ exchanged 2 and 3 H-atoms while C_6_H_8_O_10_ exchanged 1 to 4 H-atoms, while both had a sensitivity enhancement of near 1. A more consistent trend could be seen for benzene dimer products, similarly to the naphthalene system. The dimer exchanged 2, 3 or 4 H-atoms with *r* consistently decreasing as the number of exchangeable hydrogen atoms increased. Specifically, C_12_H_14_O_8_, C_12_H_14_O_9_, C_12_H_14_O_11_, and C_12_H_14_O_12_ mostly moved 2 ion mass units and had *r* equal to 1.1–1.5. On the other hand, dimers C_12_H_14_O_10_, C_12_H_14_O_12_, and C_12_H_14_O_13_ exchanged more than 2 H-atoms and their *r* was 1.0–1.1. Overall, the comparison of the two VOC systems with addition of heavy water showed that the enhancement ratio is not a good predictor for the absolute number of OH/OOH groups (hydroxy, hydroperoxy, peroxy acid or carboxylic acid groups) in the oxidation products. The higher number of oxygen atoms makes molecules more polar and hence strengthens the H-bonds with the reagent ion.^21^ This is why the *r* would rather correlate with the number of oxygen atoms than the number of exchangeable hydrogens (Fig. 6d). However, it is clear that neither the oxygen number nor the number of exchangeable H-atoms is a perfect predictor of the sensitivity enhancement ratio and hence likely also of absolute sensitivity. The potential relationship between *r* and instrumental sensitivity is further discussed in section 3.6.

### 3.5. Implications for detecting high molecular weight compounds

**Fig. 9:**
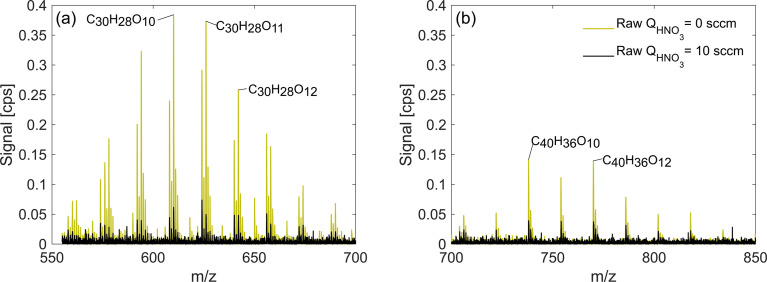
Trimers (a) and tetramers (b) observed in the high RO_2_ naphthalene experiment (exp. 2). Note that sensitivity enhancement is greater for trimers than for tetramers.

The sections above focused on exploring the change in detection sensitivities of monomers and dimeric accretion oxidation products as a response to change in the HNO_3_ concentration in the inlet. In addition, in exp. 2 and 3, we also detected adducts corresponding to accretion products of three or four oxidation product units. In these experiments, trimers and tetramers were also detected with higher signals when the HNO_3_ concentration was lowered (Fig. 9). Trimers and tetramers with corresponding formulae C_30_H_26–28_O_8–15_ and C_40_H_36_O_10–15_ are either covalently bonded products or molecular clusters. Out of all our experiments, they only appeared in high RO_2_ naphthalene runs (exp. 2 and 3) and due to the lack of variability in the elemental composition, it is plausible to assume that they are clusters. For instance, in exp. 2, C_20_H_18_O_6_ is the most abundant dimer and C_40_H_36_O_12_ is the most abundant tetramer, which is a direct sum of two of the dimers. This is also supported by the lack of detected C_20_ RO_2_ radicals needed for formation of covalently bonded accretion products. Further investigation of the origin of these products is a subject of future studies and they are referred to as clusters in the context of this study.

The trimers and tetramers in exp. 2 experienced a mean sensitivity enhancement of 5.6 and 3.2 respectively compared to 9.0 and 3.1 for monomers and dimers. Based on this result, it is clear that also very large organic clusters are not detected at a maximum sensitivity. Therefore, it is likely that their concentration could be underestimated. While it is possible that the composition of these particular clusters is not representative of real atmospheric clusters, it is very likely that by decreasing the HNO_3_ concentration in the CI-inlet we could increase the sensitivity of NO_3_^−^-CIMS to different large clusters that can form for instance during initial steps of new particle formation. The clusters in the real atmosphere are present in very small concentrations and are prone to fragmentation inside the CIMS.^33^ Increasing the sensitivity henceforth may allow for better detection, identification and characterization of ambient-relevant clusters. This hypothesis would need further exploration in the laboratory and in the field.

### 3.6. Implications for quantification

As shown above, many of the oxidation products were not detected at the collision limit in NO_3_^−^-CIMS under normal operation as their signal increased with the decreased nitric acid concentration. Even for the most oxidized naphthalene products, the measured concentrations can apparently be underestimated if the calibration factor obtained for sulfuric acid is applied. HOMs formed in naphthalene oxidation may be underestimated by at least a factor of 4, while in benzene – by a factor of 1.3 (see Table 1). Moreover, some of the molecules in naphthalene oxidation that can be determined as extremely low- or ultra-low volatility organic compounds (ELVOC or ULVOC)^25,26^ are still not detected at the collision limit. This is in contrast to the assumption used in the literature for correcting the sensitivities towards less oxygenated products when calculating the particle growth rates (*e.g.* Tröstl *et al.*^11^). While generally the volatility-based approach is physically justified, determining the relationships with absolute instrumental sensitivities may be more complicated.

We observed some relationship between the number of O-atoms, the number of exchangeable H-atoms and the enhancement of the sensitivity as a response to the lowered HNO_3_ concentration. This relationship is clearer for more oxygenated products (Fig. S1 and S2†). It is plausible to hypothesize that the exact enhancement ratio may provide information about the relative binding enthalpies between the sample molecules and NO_3_^−^. If the concentration of nitrate reagent ions, (HNO_3_)_0–2_NO_3_^−^, is much higher than that of the detected oxidation product, then the enhancement ratio should only depend on the ability of the organic oxidation product to steal the nitrate ion from the nitric acid-nitrate cluster, which in turn is dependent on the cluster formation energy. This relationship also follows computations conducted for this CIMS set-up by Hyttinen *et al.*^15^ Iyer *et al.* developed the idea further and showed the relationship between cluster binding enthalpy and absolute sensitivity for iodide-based CIMS.^34^ If the relationship between the sensitivity enhancement ratio and instrumental sensitivity is established, it will greatly aid in quantification of both HOMs and other oxidation products detected by NO_3_^−^-CIMS.

To address similar issues for iodide ion-based CIMS calibration, Lopez-Hilfiker *et al.*^35^ relied on the voltage scanning method. By increasing the voltage difference between the skimmer of the first quadrupole and the entrance to the second quadrupole, d*V*, in the collision–dissociation chamber (CDC) of the CIMS, they were able to measure *V*_50_ for each ion, a voltage value that causes the loss of 50% of the ion signal. Using modelled enthalpies for molecule–ion adducts, a calibration curve is developed for relating *V*_50_ to sensitivity. Potentially, our method with varying HNO_3_ concentrations could be equivalent in predicting the binding enthalpies of different organic oxidation products with NO_3_^−^ with the important advantage of enhanced signals, as opposite to the reduced signal. Further tests comparing these two potentially complementary methods are necessary.

Directly relating the observed enhancement ratio to the instrumental sensitivity may be challenging due to the specific limitations of the CIMS instrument. The largest challenge is that the enhancement in the signal will depend on the HNO_3_ concentration in the ionization region. In other words, the ratio will vary between different inlets or experiments conducted far apart in time. This issue could be overcome with further inlet development. For instance, an inlet with lowered wall memory effects in which HNO_3_ can be added at varying concentrations in a controlled manner could help make comparison in time and between instruments more robust. In addition, knowing the absolute HNO_3_ concentration will help in modeling the corresponding charging efficiency in such CIMS set-ups.

Another challenge in determining the sensitivities for products of organic oxidation is posed by the presence of isomers. This follows also from Fig. 7 and 8, in which some of the molecules with the same chemical formula exchanged different numbers of hydrogens with D_2_O. As a result, the sensitivity enhancement ratio for a given ion composition is a weighted sum of enhancement ratios of all of its isomers. The presence of isomers will complicate the comparison of the modeled and measured results, as well as relationships between the *r* and oxygen content, the O : C ratio and other parameters. However, this method may be helpful in estimating bulk HOM concentrations.

While Berndt *et al.*^36^ showed that nitrate ion-based CIMS is not sensitive to α- and β-pinene OH oxidation products when compared to acetate ion-based CIMS, it seems to be different in aromatic systems. Ultimately, the sensitivity is dictated by the stereochemical structure of the multifunctional product of a chain reaction, emphasizing the importance of detailing the exact oxidation mechanisms. Many of the benzene oxidation products are detected at the collision limit in excess of HNO_3_, while almost none of the naphthalene products are. However, the exact formation pathways of these compounds matter. If the amount of multigeneration oxidation is modified, the sensitivity may also be affected. It is possible that very few organic oxidation products have collision-limited sensitivity in NO_3_^−^-CIMS, and the current method can be used to gauge this effect.

## 4 Conclusions

We conducted benzene and naphthalene oxidation experiments in which we detected the oxidation products using nitrate-based CIMS at two different reagent HNO_3_ concentrations. By running chemical ionization at HNO_3_ deficit, we could detect a wider range of less oxygenated products, including early generation peroxy radicals. This can especially assist in detection of species in experiments with lower reactant concentrations. Additionally, some of the ions were only detected at a deficit of nitric acid vapor and were not detected under normal operating conditions.

With less neutral HNO_3_ in the ionization region to compete with the adduct formation with NO_3_^−^, we observed enhanced sensitivity for most of the organic species. This enhancement was larger for naphthalene than benzene oxidation products suggesting that sensitivity is lower for naphthalene products. One of the possible reasons is that the larger naphthalene oxidation products have H-bond donor groups that are further apart causing lower binding energies with NO_3_^−^. This general trend was confirmed by quantum chemical computations: the compounds with OH groups further apart had lower binding energies with NO_3_^−^. Additionally, for products with the same number of exchangeable H-atoms, naphthalene oxidation products had higher enhancement than benzene products. Other plausible reasons for lower sensitivity enhancement in the benzene system include the higher relative abundance of ring-opened products eliminating steric effects when forming adducts with NO_3_^−^ as well as the higher relative strength of the H-bond donor functional groups.

The observed enhancement (especially for naphthalene-derived compounds) has an implication on how we determine the sensitivity of NO_3_^−^-CIMS and the uncertainty of the estimated yields. For instance, HOM yields for naphthalene could be underestimated at least by a factor of 4. It is clear that some of the products that are approximated to belong to ultra-low and extremely low volatility classes are also not detected at the collision limit. No enhancement was observed for many benzene oxidation products with 7 or more oxygen atoms but only for a few most-oxygenated accretion products in the naphthalene system, meaning that these were detected at the collision limit in normal inlet operation (excess HNO_3_). This has implications on our ability to determine the composition and concentration of low-volatility products in systems with high aerosol-forming potential in the atmosphere, such as naphthalene and α-pinene. We suggest that reduction of HNO_3_ in the inlet could be used as a method for inferring similar information on sensitivity in other experiments and systems.

Our results show that not all accretion products are detected at the collision limit. For example, the sensitivity of trimer and tetramer species was enhanced by the decreased HNO_3_ concentration. This suggests that the concentrations of accretion products in the atmosphere are likely underestimated by NO_3_^−^-CIMS measurements. In addition, it is possible that by decreasing the nitric acid vapor concentration in the inlet, we can detect molecular clusters in the real atmosphere that are not observed under normal instrument operation. However, more experiments are needed to confirm this.

By checking the number of exchangeable hydrogens through introducing heavy water to the flow reactor, we show that the oxidation products that display no enhancement in the signal had exchanged 2 or more hydrogens. However, the signal of naphthalene oxidation products that exchanged even 3 or 4 hydrogens had still an enhancement of a factor of 1.5–2.2. On the other hand, some compounds with only 1 available H-bond donor were also detected, contradicting the common assumption that 2 H-bond donor groups are needed. The enhancement ratio also had a negative relationship with O : C, OSc, oxygen content and lowered volatility, though challenges exist in how to interpret the relationship between the enhancement ratio and the instrumental sensitivity.

We would like to emphasize that the ambient concentration of HNO_3_ will affect the sensitivity of the instrument for species not detected at the collision limit and should be considered when making ambient measurements as well as when applying the varying HNO_3_ method introduced in this work. Future studies can focus on developing a well-controlled nitrate inlet and testing the effect of HNO_3_ deficit in biogenic VOC oxidation systems.

## Data availability

The data supporting this article have been included as part of the ESI.†

## Author contributions

OG: conceptualization, formal analysis, investigation, methodology, visualization, writing – original draft; AK: investigation, methodology, writing – review & editing; SJ: investigation, writing – review & editing; SB: methodology, writing – review & editing; NH: writing – review & editing; SI: writing – review & editing; MR: conceptualization, formal analysis, funding acquisition, methodology, resources, supervision, writing – review & editing.

## Conflicts of interest

There are no conflicts to declare.

## Supplementary Material

EA-004-D4EA00087K-s001
