# Supplementary material for: Enhanced detection of aromatic oxidation products using NO3− chemical ionization mass spectrometry with limited nitric acid
Source: Environ Sci Atmos. 2024 Oct 15;4(12):1368–81. doi: 10.1039/d4ea00087k (PMC11505638; doi:10.1039/d4ea00087k)
Supplement: EA-004-D4EA00087K-s001 [file EA-004-D4EA00087K-s001.pdf]

Supplementary Information (SI) for Environmental Science: Atmospheres.
This journal is © The Royal Society of Chemistry 2024

Supplementary Information for

Enhanced detection of aromatic oxidation products using NO_3^- chemical ionization mass spectrometry with limited nitric acid

Olga Garmash[1,2*], Avinash Kumar[1], Sakshi Jha[1], Shawon Barua[1], Noora Hyttinen[3], Siddharth Iyer[1], Matti Rissanen[1,4*]

[1]Aerosol Physics Laboratory, Physics Unit, Faculty of Engineering and Natural Sciences, Tampere University, FI-33720, Tampere, Finland

[2]Department of Chemistry, University of Copenhagen, DK-2100, Copenhagen, Denmark

[3]Atmospheric Research Centre of Eastern Finland, Finnish Meteorological Institute, FI-70211, Kuopio, Finland

[4]Department of Chemistry, University of Helsinki, FI-00014, Helsinki, Finland

[*]corresponding authors

This file includes
Figures S1-S4
Tables S1-S4

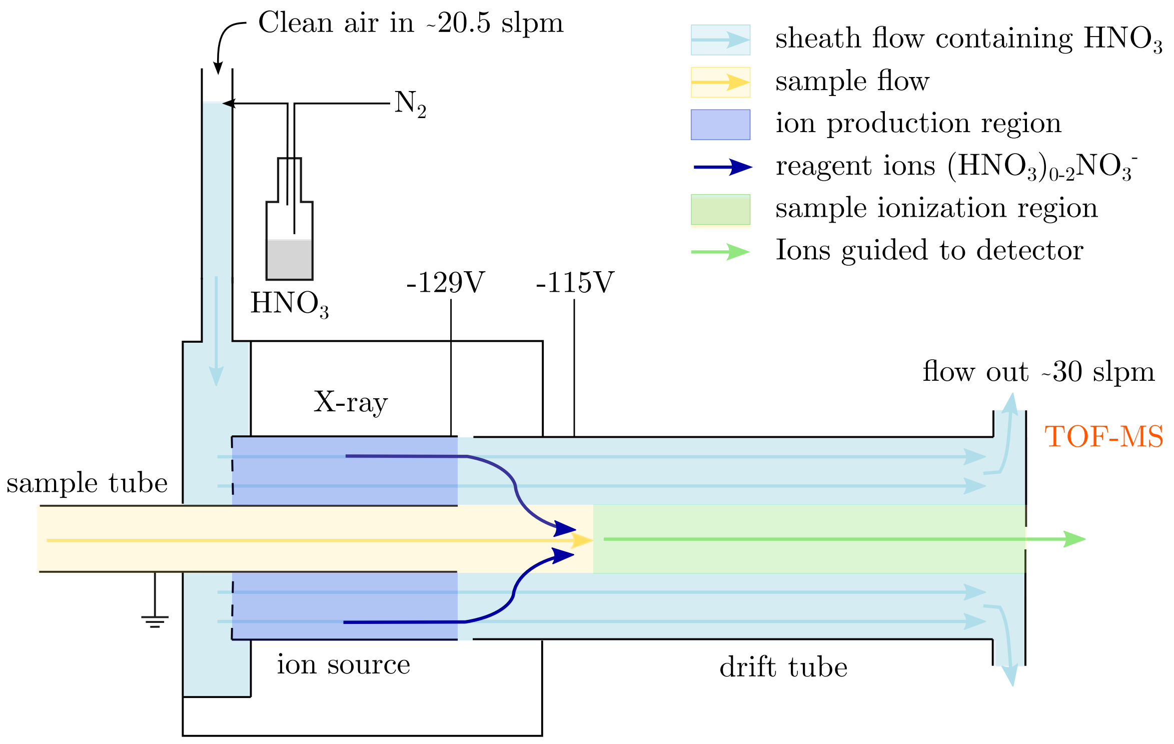

Figure S1. The schematic of Eisele-type nitrate ion chemical ionization inlet. Indicated values for flows and voltages correspond to the ones used in this study. Two regions where HNO_3 concentration can affect instrumental sensitivity are the ion production region and the sample ionization region. The flows in this schematic are approximated as ideal. In real conditions, some diffusion of neutral HNO_3 from the sheath flow into the sample ionization region may happen.

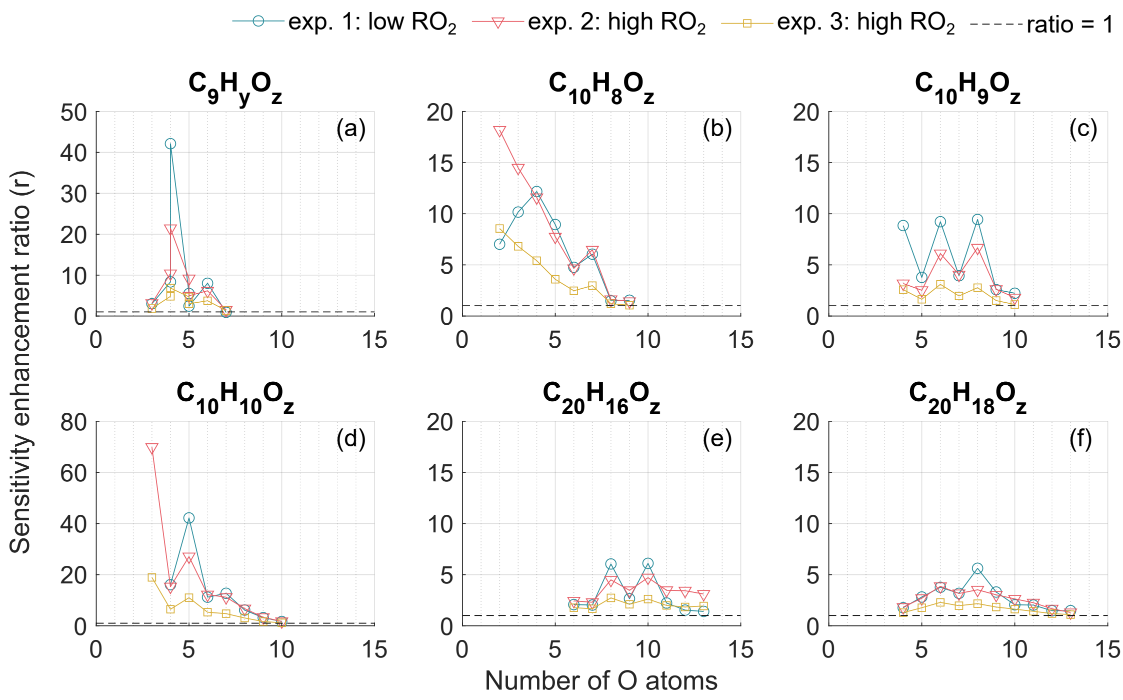

Figure S2. Enhancement in sensitivity for different product groups as function of number of oxygen atoms in the molecule in naphthalene oxidation experiments (exp. 1-3). Panels (a)-(d) show monomers and panels (e)-(f) show accretion products.

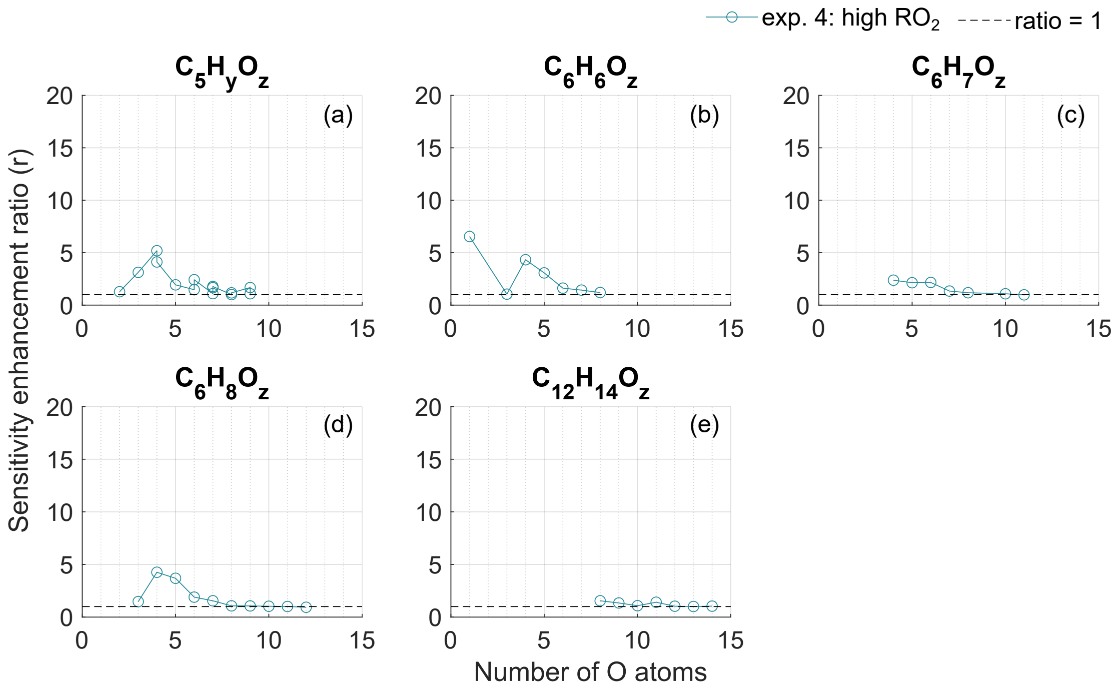

Figure S3. Enhancement in sensitivity for different molecular groups in benzene oxidation experiment (exp. 4).

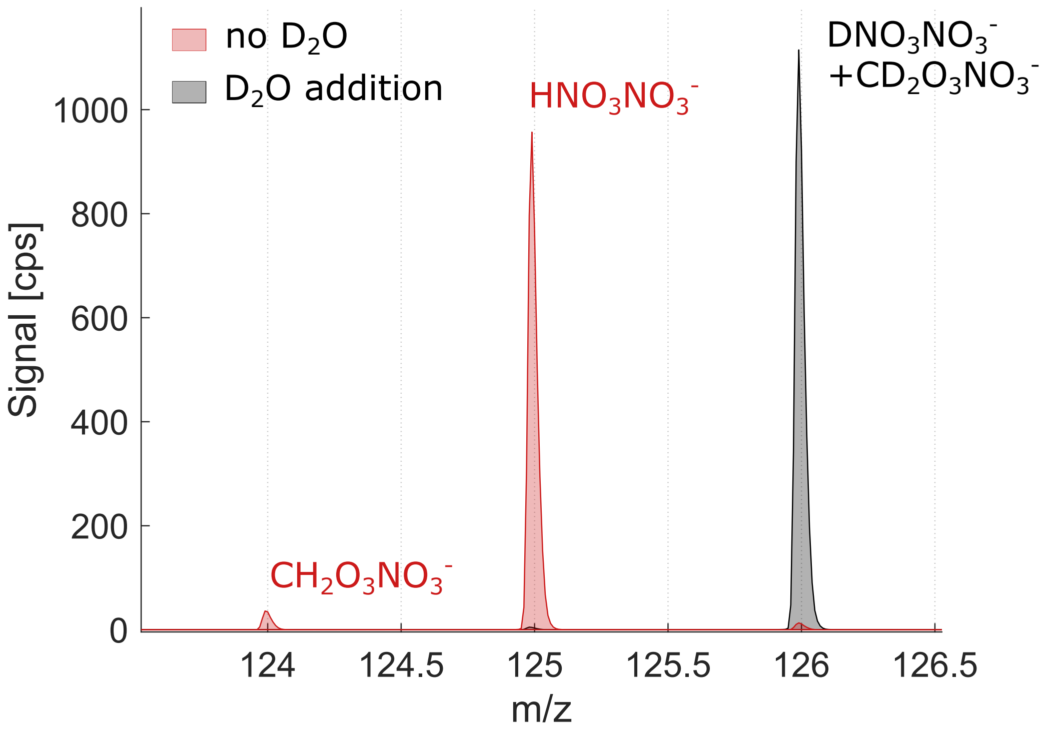

Figure S4. D_2O addition in naphthalene oxidation experiment (exp. 5) displays full conversion of HNO3NO3- to DNO3NO3-. The increase in signal at m/z 126 as compared to m/z 125 is likely due to simultaneous conversion of carbonic acid.

Table S1. Signals (counts per second) for each detected product in naphthalene experiments 1-3. 0/1 indicate the nitric acid flow settings (1=flow on 10 sccm, 0=flow off). All ions are charged negatively and reagent ion NO3 is included into the formula.

Exact mass m/z	Ion composition	Exp 1, 0	Exp 1, 1	Exp 2, 0	Exp 2, 1	Exp 3, 0	Exp 3, 1
**222.0408**	C10H8N1O5	0.76	0.11	2.42	0.13	2.59	0.30
**226.0357**	C9H8N1O6	0.28	0.09	1.61	0.52	1.97	1.07
**238.0357**	C10H8N1O6	1.63	0.16	30.63	2.11	36.23	5.32
**240.015**	C9H6N1O7	0.63	0.08	3.06	0.29	3.85	0.80
**240.0514**	C10H10N1O6	1.22	0.00	20.06	0.29	25.57	1.35
**242.0306**	C9H8N1O7	0.88	0.02	11.99	0.56	15.22	2.22
**242.067**	C10H12N1O6	0.09	0.03	0.00	0.07	0.30	0.02
**254.0306**	C10H8N1O7	2.07	0.17	23.50	2.03	29.36	5.42
**255.0385**	C10H9N1O7	0.26	0.03	1.40	0.44	1.57	0.61
**256.0463**	C10H10N1O7	4.67	0.29	83.59	5.46	105.40	16.33
**258.0255**	C9H8N1O8	0.35	0.06	5.08	0.55	6.13	1.31
**260.0412**	C9H10N1O8	0.22	0.09	1.79	0.36	2.11	0.70
**270.0255**	C10H8N1O8	4.61	0.52	71.71	9.25	87.19	24.28
**271.0334**	C10H9N1O8	1.33	0.35	4.98	1.98	5.52	3.38
**272.0412**	C10H10N1O8	9.71	0.23	125.20	4.63	157.82	14.37
**274.0204**	C9H8N1O9	3.45	0.43	51.57	8.35	60.34	16.18
**286.0204**	C10H8N1O9	3.43	0.72	44.05	9.46	53.17	21.51
**287.0283**	C10H9N1O9	1.62	0.18	5.87	0.96	5.46	1.76
**288.0361**	C10H10N1O9	2.37	0.21	86.17	7.08	106.16	20.04
**290.0154**	C9H8N1O10	0.35	0.39	12.86	8.66	15.57	11.59
**290.0518**	C10H12N1O9	0.52	0.14	6.22	2.47	9.35	5.50
**302.0154**	C10H8N1O10	13.44	2.22	294.32	45.45	343.83	115.69
**303.0232**	C10H9N1O10	0.72	0.18	3.54	0.88	4.03	2.06
**304.031**	C10H10N1O10	6.15	0.48	144.67	12.94	168.77	35.62
**318.0103**	C10H8N1O11	0.78	0.52	18.94	11.65	23.87	19.76
**319.0181**	C10H9N1O11	8.47	0.90	23.98	3.59	21.54	7.77
**320.0259**	C10H10N1O11	2.72	0.44	70.33	10.48	81.02	26.56
**334.0052**	C10H8N1O12	3.58	2.32	44.13	30.81	46.76	44.40
**335.013**	C10H9N1O12	1.56	0.60	4.23	1.62	4.31	2.85
**336.0208**	C10H10N1O12	4.68	1.47	65.50	19.63	70.33	42.35
**351.0079**	C10H9N1O13	2.81	1.26	6.04	3.36	5.54	4.93
**352.0158**	C10H10N1O13	1.45	0.87	18.28	10.67	19.49	16.83
**384.1089**	C20H18N1O7	18.50	10.48	157.49	86.38	200.91	156.29
**400.1038**	C20H18N1O8	3.63	1.29	82.87	30.79	103.68	58.76
**414.0831**	C20H16N1O9	0.23	0.11	3.86	1.59	4.90	2.78
**416.0987**	C20H18N1O9	24.45	6.50	221.31	57.38	269.13	117.74
**430.078**	C20H16N1O10	0.10	0.05	2.56	1.11	3.21	1.93
**432.0936**	C20H18N1O10	3.80	1.20	98.34	31.13	114.63	58.40
**446.0729**	C20H16N1O11	0.36	0.06	4.72	1.05	5.14	1.88
**448.0885**	C20H18N1O11	3.72	0.66	51.92	14.76	62.30	28.80
**450.0678**	C19H16N1O12	0.55	0.28	9.50	5.73	10.97	8.68
**462.0678**	C20H16N1O12	0.17	0.07	4.65	1.34	5.28	2.52
**464.0834**	C20H18N1O12	4.36	1.32	95.28	31.48	106.73	58.25
**478.0627**	C20H16N1O13	0.29	0.05	3.77	0.80	3.98	1.53

**480.0784**	C20H18N1O13	0.77	0.37	29.05	11.06	33.21	20.44
**494.0576**	C20H16N1O14	0.10	0.04	3.18	0.92	3.48	1.75
**496.0733**	C20H18N1O14	2.32	1.15	38.28	17.00	40.21	28.49
**510.0526**	C20H16N1O15	0.05	0.03	1.37	0.40	1.55	0.84
**512.0682**	C20H18N1O15	0.31	0.21	10.43	6.26	11.11	9.31
**526.0475**	C20H16N1O16	0.05	0.04	0.81	0.26	0.83	0.43
**528.0631**	C20H18N1O16	0.61	0.41	7.20	5.54	7.38	6.76

Table S2. Signals (counts per second) for trimer and tetramer accretion products in naphthalene experiment 2 (Figure 9). 0/1 indicate the nitric acid flow settings (1=flow on 10 sccm, 0=flow off). All ions are charged negatively and reagent ion NO3 is included into the formula.

**Exact mass m/z**	**Ion composition**	**Exp 2, 0**	**Exp 2, 1**
**562.1719**	C30H28N1O10	0.78	0.11
**576.1511**	C30H26N1O11	1.59	0.30
**578.1668**	C30H28N1O11	1.80	0.23
**592.1461**	C30H26N1O12	2.25	0.40
**594.1617**	C30H28N1O12	3.43	0.33
**608.141**	C30H26N1O13	2.76	0.44
**610.1566**	C30H28N1O13	4.22	0.58
**624.1359**	C30H26N1O14	3.41	0.74
**626.1516**	C30H28N1O14	4.07	0.47
**640.1308**	C30H26N1O15	2.04	0.46
**642.1464**	C30H28N1O15	2.75	0.44
**656.1257**	C30H26N1O16	2.19	0.55
**658.1414**	C30H28N1O16	1.74	0.32
**672.1206**	C30H26N1O17	0.93	0.31
**674.1363**	C30H28N1O17	1.03	0.33
**690.1312**	C30H28N1O18	0.73	0.25
**704.1105**	C30H26N1O19	0.35	0.13
**706.2294**	C40H36N1O11	0.62	0.25
**722.2243**	C40H36N1O12	0.57	0.21
**738.2192**	C40H36N1O13	1.70	0.46
**754.2141**	C40H36N1O14	1.35	0.39
**770.209**	C40H36N1O15	1.77	0.43
**786.204**	C40H36N1O16	1.05	0.36
**802.1989**	C40H36N1O17	0.62	0.24
**818.1938**	C40H36N1O18	0.57	0.20
**834.1887**	C40H36N1O19	0.27	0.10

Table S3. Signals (counts per second) for each detected product in benzene experiment (exp. 4). 0/1 indicate the nitric acid flow settings (1=flow on 10 sccm, 0=flow off). All ions are charged negatively and reagent ion NO3 is included into the formula.

Exact mass m/z	Ion composition	Exp 4, 0	Exp 4, 1
**156.0302**	C6H6N1O4	0.77	0.12
**160.0251**	C5H6N1O5	210.11	164.49
**176.0201**	C5H6N1O6	12.73	4.08
**188.0201**	C6H6N1O6	83.36	79.84
**189.9993**	C5H4N1O7	12.43	2.40
**190.0357**	C6H8N1O6	3.19	2.16
**192.015**	C5H6N1O7	29.32	7.13
**192.0514**	C6H10N1O6	0.39	0.11
**204.015**	C6H6N1O7	84.05	19.42
**205.0228**	C6H7N1O7	6.25	2.63
**206.0306**	C6H8N1O7	240.34	56.40
**208.0099**	C5H6N1O8	19.30	10.00
**220.0099**	C6H6N1O8	46.46	15.13
**221.0177**	C6H7N1O8	2.60	1.22
**222.0255**	C6H8N1O8	329.39	89.36
**224.0048**	C5H6N1O9	32.56	22.17
**224.0412**	C6H10N1O8	0.95	0.94
**226.0205**	C5H8N1O9	21.00	8.67
**236.0048**	C6H6N1O9	19.85	12.26
**237.0126**	C6H7N1O9	1.77	0.82
**238.0205**	C6H8N1O9	92.92	48.95
**239.9997**	C5H6N1O10	30.94	28.27
**241.0075**	C5H7N1O10	3.59	2.16
**242.0154**	C5H8N1O10	20.79	11.71
**251.9997**	C6H6N1O10	11.91	8.33
**253.0075**	C6H7N1O10	2.66	1.99
**254.0154**	C6H8N1O10	45.13	29.13
**255.9946**	C5H6N1O11	62.43	62.16
**258.0103**	C5H8N1O11	6.04	5.16
**267.9946**	C6H6N1O11	19.51	16.27
**269.0025**	C6H7N1O11	3.16	2.68
**270.0103**	C6H8N1O11	94.86	88.87
**271.9896**	C5H6N1O12	12.59	7.52
**274.0052**	C5H8N1O12	4.18	3.85
**286.0052**	C6H8N1O12	39.21	36.95
**300.9923**	C6H7N1O13	3.23	2.99
**302.0001**	C6H8N1O13	26.01	25.25
**316.9872**	C6H7N1O14	2.97	3.03
**317.995**	C6H8N1O14	8.61	8.52
**333.9899**	C6H8N1O15	5.81	6.23
**348.0572**	C12H14N1O11	65.33	42.18
**364.0522**	C12H14N1O12	20.24	15.09
**380.0471**	C12H14N1O13	10.17	9.39
**396.042**	C12H14N1O14	10.66	7.55
**412.0369**	C12H14N1O15	15.29	14.76

**428.0318**	C12H14N1O16	4.47	4.44
**444.0267**	C12H14N1O17	5.15	4.95

Table S4. Regression analysis coefficients presented in Figure 6 of the main text. r is the sensitivity enhancement ratio, O:C is oxygen-to-carbon ratio, OSc is oxidation state of carbon, C* is saturation vapor concentration, and nO is number of oxygen atoms (see Methods in main text for more details).

	**Exp.**	**VOC**	**Linear model**	**y**	**x**	**a**	**b**	**R^2**
**Figure 6a**	2	npt	$\log_{10}(y)= ax+b$	r	O:C	-0.59	0.97	0.10
	4	ben	$\log_{10}(y)= ax+b$	r	O:C	-0.31	0.55	0.36
**Figure 6b**	2	npt	$\log_{10}(y)= ax+b$	r	OSc	-0.33	0.72	0.12
	4	ben	$\log_{10}(y)= ax+b$	r	OSc	-0.15	0.36	0.33
**Figure 6c**	2	npt	$\log_{10}(y)= ax+b$	r	$\log_{10}(C^*)$	0.035	0.89	0.37
	4	ben	$\log_{10}(y)= ax+b$	r	$\log_{10}(C^*)$	0.027	0.27	0.43
**Figure 6d**	2	npt	$\log_{10}(y)= ax+b$	r	nO	-0.08	1.22	0.36
	4	ben	$\log_{10}(y)= ax+b$	r	nO	-0.05	0.58	0.48
